# Cardiac remodelling during pregnancy: whither the guinea pig?

**DOI:** 10.1093/cvr/cvu198

**Published:** 2014-08-25

**Authors:** Michael J. Taggart, Richard Hume, Jon Lartey, Mark Johnson, Wing-Chiu Tong, Kenneth T. Macleod

**Affiliations:** 1 Institute of Cellular Medicine, Newcastle University, 3rd floor, Leech Building Medical School, Newcastle, UK; 2 Imperial College London, London, UK

**This letter refers to ‘Pregnancy as a cardiac stress model’ by E. Chung and L.A. Leinwand, Cardiovascular Research 2014;101:561–570**

We were interested to read the recent article published by Chung and Leiwand^1^ entitled *Pregnancy as a Cardiac Stress Model*. This, and allied articles in the Review Focus on Pregnancy-mediated Heart and Vascular Disease, eloquently brought attention to an important, yet much understudied, theme. Namely, that there is much to be learnt from elucidating the remodelling responses to the cardiovascular challenge of pregnancy that may be beneficial to informing our understanding of the sequence of events leading to compensating cardiac hypertrophy and the deleterious changes beyond those which initiate and underpin progression to heart failure.

Much of the experimental data reviewed by Chung and Leiwand^1^ was derived from mouse or rat models. This is understandable for reasons of cost, accessibility, and ease of genetic manipulation that facilitate mechanistic experimentation in a timely fashion. An additional important feature of the Chung and Leiwand^1^ article was to draw distinctions between exercise-induced remodelling and pregnancy-dependent adaptations, and the majority of work in the former category has also been undertaken in rat or mouse models. This therefore offers a basis on which to compare the two physiological remodelling circumstances.

Notwithstanding these benefits, one should also recognize where limits reside for extrapolating information from mouse or rat models of cardiac function in pregnancy to the human. There are, to our minds, two issues of note. First, the cardiac remodelling events occurring in response to pregnancy are likely to be mediated, in part, by changing levels of sex steroidal hormones.^1^ Yet mice or rats differ from humans in their mode of placentation, source of steroidogenesis, circulating gestational hormone levels, and the endocrine mechanism of parturition onset.^2^ Secondly, the mouse or rat cardiac ventricular action potential is considerably shorter than that of the human. This complicates consideration of factors that may alter the shape or duration of action potentials as is likely to occur in conditions of hypertrophic cardiac remodelling. Such factors are apt to change the electrical characteristics of the myocardium in different areas and encourage arrhythmogenesis. The relative roles played by the ionic currents underlying the action potentials are subtly different in rats/mice, and this makes extrapolations to the human situation more challenging. The systems linking the excitatory event to contraction [excitation-contraction (EC) coupling] also differ from that in human. Mice and rat hearts have very high gain EC coupling whereby a relatively small Ca influx into each ventricular myocyte triggers a much larger Ca release from the sarcoplasmic reticulum (SR) that induces contraction of the muscle cells. The system is very dependent on having the SR well filled with Ca almost to the point of overload, a factor in itself that increases arrhythmogenicity in these species. In turn, therefore, rat or mouse cardiomyocyte relaxation occurs predominantly by the sequestration of cytosolic Ca by the SR Ca-ATPase with a much lesser contribution from the sarcolemmal Na–Ca exchanger. The balance of these Ca fluxes during contraction and relaxation may be rather different in human cardiac myocytes.^2^

Therefore, it would be of considerable benefit to have an animal model that showed features of pregnancy adaptations and cardiac EC coupling that were similar to the human situation. We suggest that some of these issues can be improved by considering the use of guinea pigs to study cardiac function in pregnancy. The form of placentation, levels of circulating steroids during pregnancy, the lack of maternal progesterone withdrawal preceding parturition, the shape of the ventricular cardiac action potentials, and the balance of Ca fluxes underlying contraction and relaxation in the guinea pig are each more similar to the human situation than mice or rats.^3^ Indeed, Chung and Leiwand^1^ refer to important work from the 1980s in non-pregnant guinea pigs, whereby oestradiol infusion to non-pregnant guinea pigs resulted in similar cardiovascular changes to that arising from pregnancy.^4^ This once popular model of the pregnant guinea pig has fallen out of favour probably as a result of (i) efforts in the post-genomic age being directed towards molecular and physiological phenotyping in mice and (ii) the perceived difficulty in accurately time-mating guinea pigs due to their rather long oestrous cycle of ∼20 days. The latter issue we have overcome by daily observance of animals such that we can time-mate guinea pigs, to within 24 h, and achieve ∼90% pregnancy success. In addition, the guinea pig genome has been sequenced.^5^ Consequently, we have begun to explore the molecular changes taking place in guinea pig heart during pregnancy and compare the left-ventricular transcriptional profiles between late pregnancy and non-pregnancy. In particular, we have noted increases in the expression of genes encoding several molecules that may impact upon growth and/or EC coupling. These include the β1 adrenoceptor (ADRB1), as well as the insulin receptor (INSR) and the S1P1 receptor (S1P1R) (*Figure 1*). Of note, in the guinea pig, continued β1 adrenoceptor stimulation results in cardiac hypertrophy.^6^ Thus, our preliminary data suggest that the remodelling responses to pregnancy may be similar to the, initially beneficial, adaptive responses taking place in cardiac tissue in response to pathological insult which can, in time, tip over into detrimental changes leading to heart failure. As commented by Chung and Leiwand,^1^ a feature of hearts late in pregnancy is that they may be operating on this edge of dysfunction. If so, then exploring the functional impact on EC coupling of such pregnancy-related molecular changes may reveal important clues for the future prevention and/or treatment of failing hearts.

**Figure 1:**
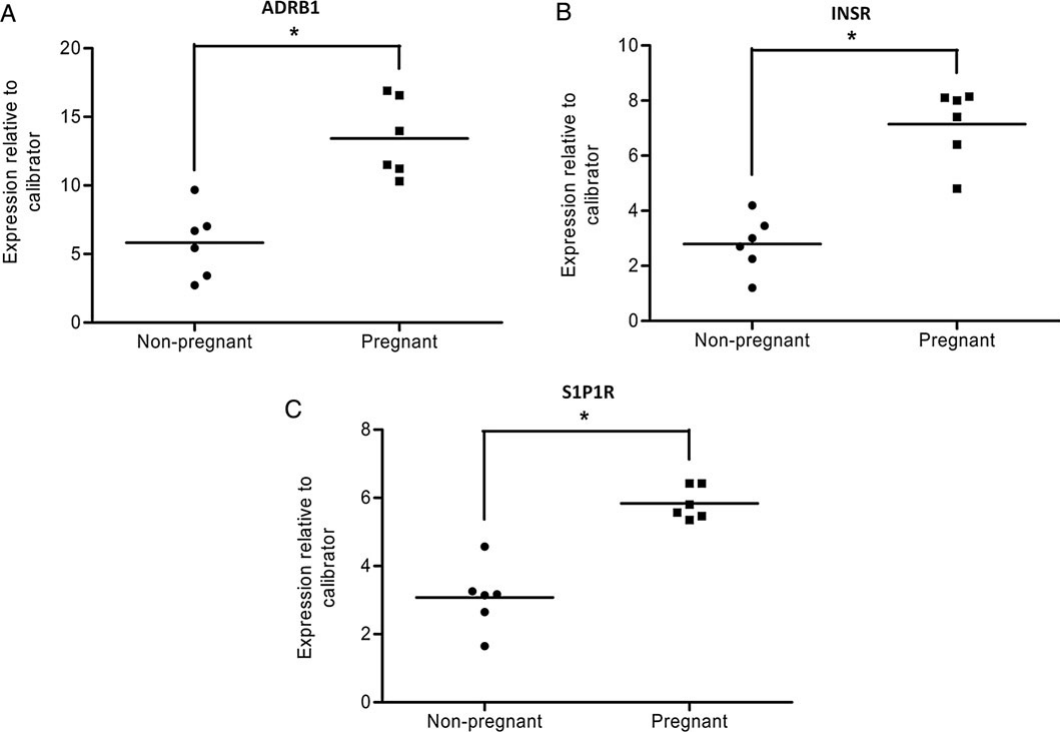
Guinea pig pregnancy induces increased expression of cardiac ventricular ADBR1, INSR, and S1P1R. RNA samples were isolated from left ventricles of non-pregnant and late pregnant (Day 68) guinea pig hearts (*n* = 6); gene-specific quantitative PCR probes were designed for (*A*) ADRB1, (*B*) INSR, and (*C*) S1P1R by alignment with the *Cavia porcellus* genome^5^ and amplicons detected by SYBR green quantification. Results are expressed as mean ±SEM fold change relative to a control RNA sample. Student’s *t*-test was used to test significance, *P < 0.05.

In summary, we add our support to the suggestion^1^ that the physiological cardiovascular remodelling of pregnancy offers a valuable situation, of scientific and clinical importance, from which we can gain insights for our understanding of, and possibly treatment for, many situations of pathological remodelling leading to heart failure. To do so with optimum impact requires us to collate data from a number of experimental animal models, accepting the limitations and benefits of each, for relevance to the human condition. As such, we suggest that the adoption of the pregnant guinea pig will provide additional valuable information for these considerations.

**Conflict of interest:** none declared.

## Funding

This work has been supported by the MRC (Bioinformatics Training fellowship to WCT, G0902091) and the BHF (PG 032/27241).
